# KIR-HLA Genotypes in HIV-Infected Patients Lacking Immunological Recovery despite Effective Antiretroviral Therapy

**DOI:** 10.1371/journal.pone.0027349

**Published:** 2011-11-03

**Authors:** Alessandro Soria, Franca Rosa Guerini, Alessandra Bandera, Elisabetta Bolognesi, Alessia Uglietti, Caterina Fusco, Patrizia Zucchi, Renato Maserati, Giuliano Rizzardini, Mario Clerici, Andrea Gori

**Affiliations:** 1 Division of Infectious Diseases, Department of Internal Medicine, San Gerardo Hospital, University of Milano-Bicocca, Monza, Italy; 2 Laboratory of Molecular Medicine and Biotechnologies, Don C. Gnocchi Foundation IRCCS, S. Maria Nascente, Milan, Italy; 3 Department of Infectious Diseases, Foundation IRCCS San Matteo Hospital, Pavia, Italy; 4 Immunohematology and Transfusion Service, A.O.R.N. A. Cardarelli, Naples, Italy; 5 Division of Infectious Diseases, Luigi Sacco Hospital, Milan, Italy; 6 University of Milan, Milan, Italy; University of Palermo, Italy

## Abstract

**Background:**

In HIV-infected individuals, mechanisms underlying unsatisfactory immune recovery during effective combination antiretroviral therapy (cART) have yet to be fully understood. We investigated whether polymorphism of genes encoding immune-regulating molecules, such as killer immunoglobulin-like receptors (KIR) and their ligands class I human leukocyte antigen (HLA), could influence immunological response to cART.

**Methods:**

KIR and HLA frequencies were analyzed in 154 HIV-infected and cART-treated patients with undetectable viral load divided into two groups: ‘immunological non responders’ (INR, N = 50, CD4^+^ T-cell count <200/mm^3^) and full responders (FR, N = 104, CD4^+^ T-cell count >350/mm^3^). Molecular KIR were typed using polymerase chain reaction-based genotyping. Comparisons were adjusted for baseline patient characteristics.

**Results:**

The frequency of *KIR2DL3* allele was significantly higher in FR than in INR (83.7% vs. 62%, *P* = 0.005). The functional compound genotype *HLA-C1^+^/KIR2DL3^+^*, even at multivariable analysis, when adjusted for nadir CD4^+^ T-cell count, was associated with reduced risk of INR status: odds ratio (95% Confidence Intervals) 0.34 (0.13−0.88), *P* = 0.03.

**Conclusions:**

Reduced presence of the inhibitory *KIR2DL3* genotype detected in INR might provoke an imbalance in NK function, possibly leading to increased immune activation, impaired killing of latently infected cells, and higher proviral burden. These factors would hinder full immune recovery during therapy.

## Introduction

Combination antiretroviral therapy (cART) has dramatically reduced morbidity and mortality in individuals infected with human immunodeficiency virus (HIV) [1]. Nevertheless, up to 30% of HIV-infected patients, despite virologically effective cART with undetectable plasma viral load, fail to achieve a satisfactory immunological recovery [2], defined as a CD4^+^ T-cell count ≥200/mm^3^. Despite been intensive investigation regarding the subject, possible mechanisms explaining this phenomenon remain largely unexplained. Low nadir CD4^+^ T-cell count, hepatitis C or other viral coinfection, age, previous AIDS-defining events, history of cART and duration of suppressive cART have all been associated with an impaired immune reconstitution [3]. More recently, higher immune activation and T-cell turnover, and higher proviral burden were found to be linked to reduced immune recovery [4]. As yet undefined host factors might explain the diversity between patients of immunological responses to cART: a role of genetic polymorphisms of cytokines [5] and human leukocyte antigen (HLA) molecules [6] has been suggested.

The impact of host genetic variation on outcome of HIV infection in the absence of antiretroviral therapy has been well documented, with recent focus on genes encoding for HLA class I molecules, whose polymorphism influences HIV control [7], [8]. The crucial role of host genetics in determining interindividual levels of protections against HIV has been further highlighted by the discovery of HLA class I as ligands for killer cell immunoglobulin-like receptors (KIR), a polymorphic set of molecules that modulate natural killer (NK) cell activity [9].

Specific epistatic interactions between HLA- and KIR-encoding genes were found to be associated with a delay in disease progression and a stronger viral control in HIV-infected cART naïve individuals [10], [11]: the functional interaction between HLA-B Bw4-Ile4 and KIR3DS1, it has been suggested, leads to activation of NK cells, T-cells or both, and to elimination of HIV-1-infected cells [12]. Modulation of receptor expression on NK cells may also play a role in the pathogenesis of HIV-1 disease progression, as recently shown by the discovery of reduced KIR2DS1 and KIR2DL1 presence on NK surfaces in advanced HIV-1 disease [13].

Prompted by these findings, we investigated whether distinct KIR-HLA genetic profiles may play a role in failed immune recovery of HIV-infected individuals treated with virologically effective cART.

## Methods

### Ethic statement and subjects

A group of 154 unrelated HIV-infected individuals were enrolled in the Infectious Diseases Unit of San Gerardo Hospital in Monza, Luigi Sacco Hospital in Milan, and San Matteo Hospital in Pavia, all the three in the Lombardy region of northern Italy. The study was approved by the review board of the three institutions (San Gerardo Hospital, Monza, Italy, Luigi Sacco Hospital, Milano, Italy, and San Matteo Hospital, Pavia, Italy). Patients participating in this study gave written informed consent according to the Declaration of Helsinki.

Inclusion criteria were stable cART for >12 months, HIV RNA <50 copies/mL over the last 6 months and written informed consent to participate to the study. On the basis of CD4^+^ responses to stable and suppressive cART, patients were divided into two groups: immunological non responders (INR), CD4^+^ ≤200/mm^3^, and full responders (FR), CD4^+^ ≥350/mm^3^. One-hundred-and-three HIV-negative subjects were evaluated as healthy controls (HC) in order to account for possible bias in the selection of HIV-infected population.

Demographic and laboratory parameters were retrieved from patients' files. Stored samples were used to perform KIR and HLA genotyping.

### HLA and KIR Genotyping

Genomic DNA was isolated from peripheral blood using standard procedures. Molecular typing of HLA B, Cw, and KIR was performed by PCR using sequence specific primers (SSP) method with commercial kits, according to the manufacturer's instructions (BAG- Lich, Germany; Invitrogen Corporation, Carlsbad, CA, USA). Detection of the alleles recognized by the specific primers was done after amplification in a GeneAmp PCR 9700 thermocycler (Applied Biosystem, Foster City, CA, USA) by gel electrophoresis on 2% agarose gel.

### KIR haplotype and ligands

Genotypes can be resolved into two broad haplotypes, termed A and B, based on KIR gene content.

The B haplotype is defined by the presence of one or more of the following genes: *2DL2*, *2DL5*, *3DS1*, *2DS1*, *2DS2*, *2DS3*, *2DS5*. If none of these are present, an AA genotype is defined.

In the A haplotype it is exceptionally rare to find variability in gene content, but there is much more allele variability. In order to classify A and/or B haplotypes we referred to the criteria adopted by Middleton on the interactive website of allele frequencies (http://www.allelefrequencies.net), which did not distinguish between AB and BB genotypes, and called any of these Bx [14], [15].

Ligands group were defined as follows:

- KIRs 2DL1 and 2DS1 have as their ligand the C2 epitope (Asparagine at position 77, Lysine at position 80) present in most of *HLA-Cw2*, *-Cw4*, *-Cw5*, *-Cw6*, *-Cw15*, *-Cw17*, *-Cw18* alleles.
- KIRs 2DL2, 2DL3 and 2DS2 have as their ligand the C1 epitope (Serine at position 77, Asparagine at position 80) present in most of *HLA-Cw1*, *-Cw3*, *-Cw7*, *-Cw8*, *-Cw12*, *-Cw14*, *-Cw16* alleles [16].
- In addition, HLA-G was considered ligand for 2DL4, HLA-Bw4 motif for 3DL1 (and 3DS1), HLA-A3 A11 for 3DL2, and HLA-Cw4 for 2DS4, as already reported; ligands for the other receptors are still unknown [14], [15].

### Statistical analysis

Differences between groups were assessed by the chi-square test for categorical variables. Fisher exact test was performed when appropriated; Student's *t* test and Mann Withney test were adopted for continuous variables, as appropriate. All statistical tests were two-sided and differences were considered statistically significant at *P*<0.05. Since the present study was largely exploratory, corrected *P* values were not reported. The association of each polymorphism with the immunological outcome (FR or INR status) was measured by the Odd Ratio (OR) and its 95% Confidence of Interval (95%CI). For each independent variable, crude and adjusted ORs and 95% CIs were calculated. In the unconditional multiple logistic regression model, including all variables associated in the crude analysis, nonsignificant variables were eliminated in a stepwise, backward elimination algorithm, to determine the final model. The significance threshold for entering a variable in the final model was set at 0.10. Analyses were performed using SAS software (version 9.1; SAS Institute, Inc., Cary, NC, USA).

## Results

### Patient characteristics

The clinical and demographic characteristics of the 154 HIV-1-infected patients are illustrated in Table 1. Fifty (32.5%) patients were listed as INR, and 104 (67.5%) were considered as FR. The two groups were similar in clinical-epidemiological and HIV-related characteristics, except for nadir CD4^+^ T-cell count, which was significantly lower in INR than FR (mean ± standard deviation: 62±56 versus 169±114 cells/mm^3^ respectively, *P*<0.0001). Noteworthy, no statistically significant differences were observed in other variables sometimes associated with reduced immunological response, such as hepatitis C coinfection, age, and years of HIV infection, while the long exposure to cART was lower for INR.

**Table 1:** Demographic and viro-immunological characteristics of 154 HIV-1-infected patients, according to immunological response to combination antiretroviral therapy.

	INR	FR	*P*-value
	N = 50	N = 104	
Age, y.	49.9 (11.4)	46.9 (9.1)	NS
Female gender, no. (%)	12 (24.0)	29 (27.9)	NS
Mode of transmission, No. (%)			NS
- IVDU	15 (30.0)	30 (28.9)	
- HETERO	27 (54.0)	55 (52.9)	
- MSM	8 (16.0)	17 (16.3)	
- OTHER/UNKNOWN	0 (0.0)	2 (1.9)	
CDC Stage, No. (%)			0.027
- A	11 (22.0)	44 (43.1)	
- B	12 (24.0)	25 (24.5)	
- C	27 (54.0)	33 (32.4)	
UNKNOWN	0 (0.0)	2 (1.9)	
Years of HIV infection	14.3 (9.4)	17.2 (6.7)	NS
Duration of cART, y.	11 (15)	16 (11)	0.045
Nadir CD4^+^ T-cell count, cells/mm^3^	62 (56)	169 (114)	<0.0001
HBV co-infection, No. (%)	3 (6.0)	7 (8.1)	NS
HCV co-infection, No. (%)	14 (28.0)	45 (43.3)	NS

Data are reported as means (standard deviation), or numbers (percentages), as appropriate.

FR  =  full responders; INR  =  immunological non responders; IVDU  =  intravenous drug users; MSM  =  men that make sex with men; HETERO  =  heterosexual; CDC  =  Center for Disease Control and Prevention; HBV  =  Hepatitis B; HCV  =  Hepatitis C; NS  =  not significant.

### HLA and KIR distribution in the cohort study

The frequencies of the different KIR genes were analyzed in HIV-infected individuals according to the two possible immunological outcomes (Table 2): results indicated that the frequency of inhibitory *KIR2DL3* allele was significantly higher in FR than INR (83.7% vs. 62.0%, *P* = 0.005). Frequency of *KIR2DS2* (an activating KIR which has been found in strong linkage disequilibrium with *KIR2DL2*) was found to be statistically higher in INR than in FR (74.0% versus 54.8%, *P* = 0.03). No statistically significant differences in frequency of KIR haplotypes (AA and Bx) were detected between INR and FR. Notably, KIR genotype frequencies were comparable between HIV-infected subjects and HC (Table 2).

**Table 2:** KIR alleles and haplotype frequencies distribution in full responders (FR), immunological non responders (INR), and in healthy controls as reference (HC). Analysis is adjusted for nadir CD4^+^ T-cell counts.

KIR gene	INR	FR	crude		adjusted		HC
	no. (%) (*N* = 50)	no. (%) (*N* = 104)	OR (95% CI)	*P*	OR (95% CI)	*P*	no. (%) (*N* = 103)
KIR2DL1	47 (94.0)	100 (96.2)	0.63 (0.11–3.70)	0.60			101 (98.1)
KIR2DL2	28 (56.0)	57 (54.8)	1.05 (0.50–2.19)	0.97			60 (58.3)
KIR2DL3	31 (62.0)	87 (83.7)	0.32 (0.14–0.74)	0.005	0.30 (0.12–0.74)	0.009	91 (88.3)
KIR2DL4	47 (94.0)	104 (100)	ND	0.03	ND	0.98	101 (98.1)
KIR2DL5A	24 (48.0)	37 (35.6)	1.57 (0.80–3.51)	0.19			41 (39.8)
KIR2DL5B	20 (40.0)	44 (42.3)	0.91 (0.43–1.91)	0.92			30 (29.1)
KIR2DS1	26 (52.0)	37 (35.6)	1.96 (0.94–4.12)	0.07			48 (46.6)
KIR2DS2	37 (74.0)	57 (54.8)	2.35 (1.06–5.27)	0.03	1.93 (0.84–4.46)	0.12	59 (57.3)
KIR2DS3	14 (28.0)	38 (36.5)	0.68 (0.30–1.49)	0.38			42 (40.8)
KIR2DS4*001/002	12 (24.0)	41 (39.4)	0.49 (0.21–1.10)	0.08			16 (15.5)
KIR2DS4*003-006	37 (74.0)	70 (67.3)	1.38 (0.61–3.15)	0.51			52 (50.5)
KIR2DS5	21 (42.0)	31 (29.8)	1.71 (0.80–3.65)	0.18			32 (31.1)
KIR3DL1	46 (92.0)	97 (93.3)	ND	0.75			97 (94.2)
KIR3DL2	49 (98.0)	104 (100)	ND	0.32			103 (100)
KIR3DL3	50 (100)	104 (100)	ND	ND			103 (100)
KIR3DS1	21 (42.0)	34 (32.7)	1.49 (0.70–3.17)	0.34			43 (41.7)
KIR2DP1	48 (96.0)	100 (96.2)	ND	1.0			102 (99.0)
KIR3DP1*001/002/004	4 (8.0)	20 (19.6)	0.36 (0.12–1.11)	0.11			14 (13.6)
KIR3DP1*003	43 (86.0)	94 (90.4)	0.65 (0.21–2.06)	0.59			100 (97.1)
**Haplotype**							
AA	8 (18.2)	35 (34.0)	0.43 (0.16–1.10)	0.08			25 (24.3)
Bx	36 (81.8)	68 (66.0)	2.32 (0.91–6.07)	0.08			78 (75.7)

**NOTE**. Data are no. (%) of participants unless otherwise specified. Statistical significance is indicated by a P value of <0.05. OR, Odds ratio; CI, Confidence Intervals.

The distribution of different HLA-B and HLA-C alleles and ligands showed that HLA-Cw*04:01 was less frequent in INR than FR (9% vs. 18.8% respectively, *P* = 0.04), while all the other alleles and ligands distributed without significant differences among the two groups (Table 3).

**Table 3:** Distribution of HLA B and C allele frequencies between immunological non responders (INR) and full responders (FR).

HLA	INR			FR			*P*
	no.	%	h	no.	%	h	
B*07:02	6	6.0		12	5.8		0.86
B*08:01	5	5.0		12	5.8		0.99
B*13:01	2	2.0		6	2.9		1
B*13:02	0	0.0		2	1.0		1
B*14:01	0	0.0		1	0.5		1
B*14:02	3	3.0		6	2.9		1
B*15:01	5	5.0		3	1.4		0.1
B*15:09	0	0.0		1	0.5		1
B*15:10	0	0.0		1	0.5		1
B*15:17	2	2.0		2	1.0	1	0.59
B*15:18	0	0.0		1	0.5		1
B*15:24	0	0.0		1	0.5		1
B*18:01	11	11.0		20	9.6	1	0.86
B*18:07	0	0.0		1	0.5		1
B*27:01	2	2.0		5	2.4		1
B*35:01	14	14.0	1	32	15.4	5	0.88
B*35:02	2	2.0		1	0.5		0.25
B*37:01	3	3.0		2	1.0		0.33
B*38:01	8	8.0		11	5.3		0.50
B*39:01	1	1.0		7	3.4		0.44
B*40:01	1	1.0		3	1.4		1
B*40:02	1	1.0		1	0.5		0.55
B*40:08	1	1.0		0	0.0		0.33
B*41:01	2	2.0		0	0.0		0.10
B*44:01	0	0.0		1	0.5		1
B*44:02	3	3.0		13	6.3		0.35
B*44:03	3	3.0		4	1.9		0.68
B*45:01	1	1.0		1	0.5		0.55
B*48:01	1	1.0		1	0.5		0.55
B*49:01	3	3.0		12	5.8		0.40
B*50:01	2	2.0		5	2.4		1
B*51:01	8	8.0		16	7.7		0.89
B*51:04	0	0.0		2	1.0		1
B*52:01	1	1.0		3	1.4		1
B*53:01	1	1.0		2	1.0		1
B*55:01	2	2.0		2	1.0		0.59
B*56:01	0	0.0		1	0.5		1
B*57:01	6	6.0		5	2.4		0.18
B*58:01	0	0.0		7	3.4		0.10
Cw*01:02	3	3.0		5	2.4		0.71
Cw*02:01	0	0.0		2	1		1
Cw*02:02	3	3.0		7	3.4		1
Cw*03:02	4	4.0		2	1.0		0.09
Cw*03:03	5	5.0		5	2.4		0.30
Cw*03:04	3	3.0		3	1.4		0.39
Cw*04:01	9	9.0		39	18.8	5	0.04
Cw*05:01	4	4.0		13	6.3		0.59
Cw*06:02	12	12.0	1	22	10.6	2	0.86
Cw*07:01	16	16.0	1	33	15.9	3	0.89
Cw*07:02	5	5.0		16	7.7	4	0.52
Cw*07:03	1	1.0		1	0.5		0.55
Cw*07:04	3	3.0	1	6	2.9	2	1
Cw*08:01	4	4.0		9	4.3		1
Cw*12:02	11	11.0	2	10	4.8	1	0.08
Cw*12:03	6	6.0		14	6.7		0.99
Cw*12:04	0	0.0		1	0.5		1
Cw*14:01	0	0.0		1	0.5		1
Cw*14:02	1	1.0		4	1.9		1
Cw*15:02	5	5.0		3	1.4		0.12
Cw*16:01	2	2.0		11	5.3	1	0.23
Cw*16:02	1	1.0		1	0.5		0.55
Cw*17:01	2	2.0		0	0.0		0.10
C1	63	63.0		121	58.2		0.49
C2	37	37.0		87	41.8		
Bw4	43	43.0		93	44.7		0.87
Bw6	57	57.0		115	55.3		

**NOTE**. Data are expressed as number (no.) of alleles and percentages (%) calculated on the total number of alleles per group (INR** = **100 alleles; FR** = **208 alleles). For each allele, the number of homozygous (h) subjects were reported. Statistical significance is indicated by a P value of <0.05.

We subsequently analyzed the different interactions of all KIR genes with their HLA-B and HLA-C ligands (Table 4). The co-presence of KIR and its respective KIR-ligand in the same individual genotype is referred to as “functional compound genotype”. Interestingly, the functional compound genotype *HLA-C1^+^/KIR2DL3^+^* is more frequent in full responders: 65.4% vs. 54%, *P* = 0.02. In contrast, the functional compound genotype *HLA-C1^+^/KIR2DL2^+^* was found to be more frequent in immunological non responders, although the difference was not statistically significant (52% vs. 44.2%, *P* = 0.88). Considering the possibility of multi-allelic expression, we analyzed the distribution of different combinations of functional compound genotype between INR and FR: we found a lower frequency of *HLA-C1^+^/KIR2DS2^-^/KIR2DL3^+^* in INR with respect to FR: 10% vs. 34.6%, OR 0.15, 95%CI 0.04-0.57, *P* = 0.003.

**Table 4:** Distribution of KIR/KIR-ligands co-presence in full responders (FR) and immunological non responders (INR). Analysis is adjusted for nadir CD4^+^ T-cell counts.

KIR/KIR-ligands	INR		FR		crude		adjusted	
	no	%	no	%	*P*	OR (95%CI)	*P*	OR (95%CI)
**2DS1^+^/C2^+^**	18	36.0	20	19.2	0.02	3.02 (1.16–7.96)	0.04	2.84 (1.04–7.76)
**2DS1^−^/C2^+^**	14	28.0	47	45.2				
**2DS1^+^/C2^−^**	8	16.0	17	16.3	0.85	0.94 (0.26–3.37)		
**2DS1^−^/C2^−^**	10	20.0	20	19.2				
**2DL1^+^/C2^+^**	30	60.0	66	63.5	0.24	0.23 (0.01–3.39)		
**2DL1^−^/C2^+^**	2	4.0	1	1.0				
**2DL1^+^/C2^−^**	17	34.0	34	32.7	1.0	ND		
**2DL1^−^/C2^−^**	1	2.0	3	2.9				
**2DL2^+^/C1^+^**	26	52.0	46	44.2	0.88	1.13 (0.51–2.51)		
**2DL2^−^/C1^+^**	19	38.0	38	36.5				
**2DL2^+^/C1^−^**	2	4.0	11	10.6	0.64	ND		
**2DL2^−^/C1^−^**	3	6.0	9	8.7				
**2DL3^+^/C1^+^**	27	54.0	68	65.4	0.02	0.35 (0.15–0.85)	0.03	0.34 (0.13–0.88)
**2DL3^−^/C1^+^**	18	36.0	16	15.4				
**2DL3^+^/C1^−^**	4	8.0	19	18.3	0.36	ND		
**2DL3^−^/C1^−^**	1	2.0	1	1.0				
**2DS2^+^/C1^+^**	35	70.0	46	44.2	0.02	2.89 (1.19–7.18)	0.06	2.41 (0.95–6.12)
**2DS2^−^/C1^+^**	10	20.0	38	36.5				
**2DS2^+^/C1^−^**	2	4.0	11	10.6	0.64	ND		
**2DS2^−^/C1^−^**	3	6.0	9	8.6				
2DS4*001^+^/Cw*04^+^	0	0.0	13	12.5	0.04	ND		
2DS4*001^−^/Cw*04^+^	9	18.0	21	20.2				
2DS4*001^+^/Cw*04^−^	12	24.0	28	26.9	0.39	1.56 (0.63–3.88)		
2DS4*001^−^/Cw*04^−^	29	58.0	42	40.4				
2DS4*003^+^/Cw*04^+^	8	16.0	21	20.2	0.83	ND		
2DS4*003^−^/Cw*04^+^	1	2.0	13	12.5				
2DS4*003^+^/Cw*04^−^	29	58.0	49	47.1	0.89	1.04 (0.41–2.63)		
2DS4*003^−^/Cw*04^−^	12	24.0	21	20.2				
**3DL1^+^/Bw4^+^**	30	60.0	63	60.6	0.68	0.63 (0.11–3.86)		
**3DL1^−^/Bw4^+^**	3	6.0	4	3.8				
**3DL1^+^/Bw4^−^**	16	32.0	33	31.7	1.00	ND		
**3DL1^−^/Bw4^−^**	1	2.0	3	2.9				
**3DS1^+^/Bw4^+^**	13	26.0	19	18.3	0.37	1.64 (0.62–4.32)		
**3DS1^−^/Bw4^+^**	20	40.0	48	46.2				
**3DS1^+^/Bw4^−^**	8	16.0	15	14.4	0.94	1.24 (0.33–4.63)		
**3DS1^−^/Bw4^−^**	9	18.0	21	20.2				
								
**C1^+^/2DL2^+^/2DL3^+^**	13	26.0	32	30.8	0.04	0.16 (0.02–1.14)	0.06	0.15 (0.02–1.13)
**C1^+^/2DL2^−^/2DL3^−^**	5	10.0	2	1.9				
**C1^+^/2DL2^−^/2DL3^+^**	14	28.0	36	34.6	0.13	0.42 (0.14–1.24)		
**C1^+^/2DL2^+^/2DL3^−^**	13	26.0	14	13.5				
**C1^+^/2DS2^+^/2DL3^+^**	22	44.0	32	30.8	0.22	0.28 (0.03–1.83)		
**C1^+^/2DS2^−^/2DL3^−^**	5	10.0	2	1.9				
**C1^+^/2DS2^−^/2DL3^+^**	5	10.0	36	34.6	0.003	0.15 (0.04–0.57)	0.01	0.16 (0.04–0.64)
**C1^+^/2DS2^+^/2DL3^−^**	13	26.0	14	13.5				

**NOTE**. Data are numbers (no.) and percentages (%) of participants unless otherwise specified. Statistical significance is indicated by a P value of <0.05.

The presence of KIR-ligand genotype is considered ^+^ when observed in at least one allele: C1^−^  =  C1^−/−^; C1^+^  =  C1^+/+^ or C1^+/−^; C2^−^  =  C2^−/−^; C2^+^  =  C2^+/+^ or C2^+/−^; Cw*04^−^  =  Cw*04^−/−^; Cw*04^+^  =  Cw*04^+/+^ or Cw*04^+/−^; Bw4^−^  =  Bw4^−/−^; Bw4^+^  =  Bw4^+/+^ or Bw4^+/−^.

OR, Odds ratio; CI, Confidence Intervals; ND, not determined.

### Multivariable analysis

On the basis of univariate analysis, a logistic regression model was performed considering immunological outcome (INR versus FR) as dependent variable, and the presence of specific KIR alleles or KIR-HLA compound genotypes as covariates. The analysis was adjusted for baseline patient characteristics and is reported in Tables 2 and 4.

Results indicated that the *KIR2DL3* allele is independently associated with a reduced risk of immunological non responder status, also after adjusting for nadir CD4^+^ T-cell count: OR (95%CI) 0.30 (0.12−0.74), *P* = 0.009. The association seemed also maintained in the functional compound genotype *HLA-C1^+^/KIR2DL3^+^*: OR (95%CI) 0.34 (0.13−0.88), *P* = 0.03.

A low nadir CD4^+^ T-cell count is an intrinsic characteristic of INR; beyond adjusting for it in the multivariable analysis, we also performed a supplementary analysis by comparing INR and FR with similar nadir CD4^+^ counts (mean ± standard deviation: 62±56 cells/mm^3^ in 50 INR versus 79.8±76.1 cells/mm^3^ in 34 FR, respectively, *P*>0.05): by excluding all the FR with a high nadir CD4^+^ counts, we still detected a lower frequency of *KIR2DL3* in INR as compared to FR (62% versus 85.3% respectively, *P*<0.05).

## Discussion

In this study, we investigated for the first time the association between KIR polymorphism and the magnitude of CD4^+^ recovery in treated HIV-infected individuals with full virologic control. Interestingly, we found that the presence of *KIR2DL3* allele has a strong “protective effect” against becoming an immunological non responder.

The mechanisms which hinder full immune restoration in HIV-infected cART-treated patients remain unclear. Previous studies focused on impaired homeostasis of T-cell adaptive immunity, leading to a more rapid T-cell turnover and apoptosis in immunological non responders [17]. The trigger for this process is still poorly defined, and possibly involves the HIV proviral burden.

The influence that genetic factors have in natural control of HIV disease in untreated individuals suggests a possible role of innate immunity in determining the magnitude of immunological recovery after successful cART. In our work, we tested this hypothesis by comparing the frequencies of different KIR and HLA polymorphisms in immunological non responders and full responders.

Despite the small numbers, the two groups are quite homogeneous for demographic and HIV-related characteristics. The only strong significant difference was, predictably, in nadir CD4^+^ T-cell count. It is well known, in fact, that low CD4^+^ nadir is predictive of incomplete immune recovery [18], [19]. The two groups differ also for the duration of antiretroviral therapy, but, at the same time, all the patients have been treated for a mean of more than ten years; therefore, the possibility that a small difference in long cART duration could influence the magnitude of immune recovery seems negligible.

By comparing the frequencies of all the identified KIR and HLA alleles, we observed that *KIR2DL3* was associated with a reduced risk of becoming an immunological non responder to cART; notably, this strong and significant association was maintained after adjusting for nadir CD4^+^ T-cell count.

*KIR2DL3* and *KIR2DL2* are different alleles at the same locus: both are inhibitory KIRs, but KIR2DL3 has a weaker inhibitory potential [20]. KIR2DL3's weaker inhibition may result in greater activation of NK cells and, consequently, in more efficient control of viral infection. The potency of KIR2DL3 varies according to the HLA-C subtypes expressed. In our work, the “protective effect” of *KIR2DL3* against becoming an immunological non responders is evident also when we considered the combined KIR/KIR-ligand genotype, in this case *HLA-C1*. Also, an additional explanation for consideration is that the virus could have accumulated escape variants in certain HLA-C restricted epitopes (drug or T-cell driven), leading to a loss of HLA-C binding to KIR2DL3. This could also lead to increased NK cell killing. We do not have information on viral sequences, but this might prove an interesting follow-up analyses.

Similar results have been found in another model of treatment of a persistent viral infection, hepatitis C (HCV): the presence of *KIR2DL3* was associated with better response to anti-HCV treatment [21].

Our findings support a role of the *KIR2DL3* allele in producing sustained immune recovery, although the corresponding mechanisms could be only postulated. Recently, it has been suggested that NK cell loss during SIV infection in pigtail macaques could favour gut damage and microbial translocation, thus resulting in rapid disease progression [22]. Microbial translocation seems also to influence the magnitude of immunological recovery in treated individuals [23]. Therefore we could speculate that weaker NK activity at mucosal level (a result of the balance of inhibitory and activating KIRs) is associated with increased gut damage, and subsequent microbial translocation and immune activation. Higher levels of immune activation have been described in immunological non responders [4], and are associated with impaired T-cell homeostasis [23]. Additional functional studies will be necessary to clarify this hypothesis.

Another mechanism possibly hindering full immune recovery might involve NK cells in their role of killing latently HIV-infected cells. NK cells kill ‘missing self’ targets as a result of the balance between inhibitory and activating signals transmitted by different KIR molecules [24]. Thus, it is possible to speculate that reduced killing capacity (due to decreased presence of KIR2DL3) is associated with a higher proviral DNA burden in peripheral blood mononuclear cells, which in turn has been associated with incomplete immune restoration in immunological non responders [17].

Finally, we could hypothesize that the preservation of competent NK cells, through a favorable equilibrium between activating and inhibitory KIRs, might promote CD4^+^ T-cell recovery (via dendritic cells or directly through cytokine production).

Our study has some important limitations: due to the exploratory nature of the analysis, we did not apply any adjustment for multiple comparisons. However, our initial findings on the different distribution of *KIR2DL3* between INR and FR prompt to test this hypothesis on a larger cohort.

Another drawback of the study is that the two groups differ for nadir CD4^+^ T-cell counts, which is strongly associated with INR status. All the other characteristics were fairly similar in the two groups: of note, time of exposure to cART was quite long and not so different between INR and FR, thus suggesting that INRs were ‘true’ immunological non responders and FR were ‘true’ full responders, irrespective of their baseline CD4^+^ T-cell counts. In order to ascertain the possible role of nadir CD4^+^ T-cell counts as a confounder, we adjusted our analysis in a multivariate model, and we still found that *KIR2DL3* allele is reduced in INR as compared to FR. Moreover, when we consider only patients with low nadir CD4^+^ T-cell counts in the two groups, we found no difference in nadir CD4^+^ counts, and the frequency of *KIR2DL3* allele remains significantly lower in INR than in FR.

Our study, although hypothesis-generating, lacks functional assays to verify the proposed mechanisms. However, these preliminary findings are the first to suggest a possible link between KIR polymorphism and the magnitude of immunological response to cART in HIV infection, opening the way to new research directions in this field.

In conclusion, the reduced presence of the inhibitory *KIR2DL3* genotype in INR could be associated with a higher frequency of other KIR genotypes with stronger inhibitory potential, such as *KIR2DL2*. The observed reduction of inhibitory KIR likely results in increased immune activation; impaired killing of latently infected cells; and a higher proviral burden. These factors can justify incomplete immune recovery during therapy. The presence of different combinations of KIR inhibitory and activating genes – and their interaction with HLA – thereby warrants further evaluation, since it could prove useful as a predictor of immunological response to fully virologically effective cART.
